# Bayesian Forecasting of Bounded Poisson Distributed Time Series

**DOI:** 10.3390/e26010016

**Published:** 2023-12-22

**Authors:** Feng-Chi Liu, Cathy W. S. Chen, Cheng-Ying Ho

**Affiliations:** Department of Statistics, Feng Chia University, Taichung 40724, Taiwan; chenws@mail.fcu.edu.tw (C.W.S.C.); date880602@gmail.com (C.-Y.H.)

**Keywords:** ordinal time series data, zero-one-inflated, bounded Poisson distribution, integer-valued GARCH model, air quality index

## Abstract

This research models and forecasts bounded ordinal time series data that can appear in various contexts, such as air quality index (AQI) levels, economic situations, and credit ratings. This class of time series data is characterized by being bounded and exhibiting a concentration of large probabilities on a few categories, such as states 0 and 1. We propose using Bayesian methods for modeling and forecasting in zero-one-inflated bounded Poisson autoregressive (ZOBPAR) models, which are specifically designed to capture the dynamic changes in such ordinal time series data. We innovatively extend models to incorporate exogenous variables, marking a new direction in Bayesian inferences and forecasting. Simulation studies demonstrate that the proposed methods accurately estimate all unknown parameters, and the posterior means of parameter estimates are robustly close to the actual values as the sample size increases. In the empirical study we investigate three datasets of daily AQI levels from three stations in Taiwan and consider five competing models for the real examples. The results exhibit that the proposed method reasonably predicts the AQI levels in the testing period, especially for the Miaoli station.

## 1. Introduction

Time series of counts are non-negative integer chronological data that are widely investigated in various fields, such as epidemiology, economics, meteorology, and crime. These applications follow Zeger [1] who presented a log-linear regression model to analyze the time series of polio cases in the United States. The literature commonly uses Poisson or binomial regression models to accommodate the characteristic of integer-valued data, but such models are no longer suitable once we deal with time series datasets. Zhu et al. [2] proposed a mixture integer-valued autoregressive conditional heteroscedastic (INARCH) model to deal with computer-aided dispatch calls data. After Ferland et al. [3] proposed the integer-valued generalized autoregressive conditional heteroscedastic (INGARCH) model for time series of counts, numerous extensions and applications of INGARCH models have emerged. Notable examples include those in Weiß [4], Fokianos et al. [5], Zhu [6], Chen and Lee [7], and Chen and Lee [8]. Specifically, Xu et al. [9] introduced a new dispersed INARCH model to examine the time series of dengue cases in Singapore. Chen and Lee [8] investigated the causal relationship between climate and criminal behavior using INGARCHX models to reflect one or more exogenous series. Moreover, Chen and Khamthong [10] proposed nonlinear INGARCHX models for weekly dengue case counts in Thailand, incorporating two climatological covariates: temperature and precipitation.

Many time series of counts often have a large number of zero values like when dealing with rare diseases or rare events, resulting in excessive series dispersion. Therefore, a large strand of the literature discusses the zero-count (zero-inflation) approach to capturing such phenomena. Lambert [11] presented a count data model, the zero-inflated Poisson (ZIP) model, whose observed values are random events with a large number of zero count data in a unit of time. The ZIP model is a mixed model consisting of a Poisson assignment and zero probability, which are commonly used in quality control and accidents (e.g., Wang [12], Yau et al. [13], and Jazi et al. [14]).

There is another type of integer-valued time series, called categorical time series. Forecasting bounded ordinal time series data is challenging due to their discrete and constrained nature. Bounded ordinal data refer to data with a natural order, but limited to a specific range, such as survey responses on a scale from 1 to *K*, where K>2 . One example is the air quality index (AQI), in which the AQI value is divided into six levels, and the data are mostly between levels 1 and 2. Related research on AQI by Chen and Chiu [15] and Liu et al. [16] indicates that AQI data belong to bounded time series data. The time series of AQI levels are ordinal, because the class increases as the AQI interval value increases.

Unlike the classical Poisson distribution and the zero-inflated Poisson (ZIP) distribution, the zero-one-inflated bounded (ZOB) Poisson distribution model, as proposed by Liu et al. [16], is finite across states 0,1,…,K . This makes it more suitable for fitting restricted states to match the levels of the data. Additionally, Weiß and Jahn [17] introduced soft-clipping binomial INGARCH models as time series models for bounded counts, which also accommodate negative autocorrelations. Liu et al. [18] presented a review of the developments in INGARCH models over the past five years, focusing on unbounded and bounded non-negative counts.

The ZOB Poisson distributed model is bounded and primarily concentrated on the categories of state 0 and state 1, which occur with larger probabilities compared with other categories. The ZOB Poisson distribution to study normalcy-dominant ordinal time series is as follows:

(1) $$\begin{array}{c} P\left(Y=k\right)=\pi_{1}I_{\left\{k=0\right\}}+\pi_{2}I_{\left\{k=1\right\}}+\left(1-\pi_{1}-\pi_{2}\right)\frac{\lambda^{k}/k!}{\sum_{i=0}^{K}\lambda^{i}/i!},\;\;k=0,1,\ldots ,K, \end{array}$$

where $\pi_{1}\geq 0$ and $\pi_{2}\geq 0$ are the inflated parameters for states 0 and 1. The constraint $\pi_{1}+\pi_{2}<1$ , λ>0 , is the intensity parameter, and the integer K≥2 is a given upper bound.

In the ZOB Poisson distribution, the case of $\left(\pi_{1},\pi_{2}\right)=\left(0,0\right)$ is called a bounded Poisson distribution. When $\left(\pi_{1},\pi_{2}\right)\neq \left(0,0\right)$ , the phenomenon of inflation that may occur is in state 0 or state 1, thus allowing the datapoint to fit a normalcy-dominant ordinal time series with two possible normal states, 0 and 1. In this ZOB Poisson autoregressive (ZOBPAR) model, the intensity function $\lambda_{t}$ adopts an autoregressive structure so that $\lambda_{t}$ varies with time.

Instead of employing the method of maximum likelihood estimator as in Liu et al. [16], the present study uses Bayesian inference with the Markov chain Monte Carlo (MCMC) method to estimate unknown parameters. The advantages of using Bayesian methods include: (1) allowing the incorporation of prior knowledge or beliefs to form a prior distribution that more accurately describes the uncertainty of parameter estimates; (2) enabling simultaneous analysis of all unknown parameters and forecasting; and (3) providing probabilities that parameters fall within credible intervals, which offer a more intuitive and direct way to understand and communicate the uncertainty of parameter estimates.

This study makes three contributions to the existing literature. (1) We incorporate exogenous variables to develop the ZOBPARX model, thus accommodating more flexible situations. (2) We employ Bayesian parameter estimation methods for quantifying uncertainty. (3) We predict one-step-ahead categories for out-of-sample forecasts. The aim is to demonstrate that the model from the ZOBPARX family can effectively capture the dynamic relationships in ordinal data and provide reasonable predictions for the out-of-sample (test) period. To our knowledge, no Bayesian approach or forecast evaluation is currently available for this proposed model.

This paper proceeds as follows. Section 2 reviews the methodologies, the MCMC sampling scheme, and forecasting method. Section 3 explains the results of simulation studies and the accuracy of estimates. Section 4 provides an empirical study of AQI-level forecasts and evaluates forecast accuracy. Finally, Section 5 offers concluding remarks.

## 2. ZOBPAR and ZOBPARX Models

We denote the bounded Poisson distribution by $P^{*}\left(\lambda ,K\right)$ , where λ is the mean of Poisson, and *K* is an upper bound of category. The bounded Poisson distribution equals the ZOB Poisson distribution in Equation (1) with $\left(\pi_{1},\pi_{2}\right)=\left(0,0\right)$ . Let $D_{t}$ be an independent and identically distributed (i.i.d.) sequence having the following probability distribution:

(2) $$\begin{array}{c} P\left(D_{t}=0\right)=\pi_{1},\;P\left(D_{t}=1\right)=\pi_{2},\;P\left(D_{t}=2\right)=1-\pi_{1}-\pi_{2}. \end{array}$$

From the definition by Liu et al. [16], the ordinal time series data $Y_{t}$ are then said to follow a ZOBPAR model if:

$$\begin{array}{c} Y_{t}=\left(2-D_{t}\right)D_{t}+\left(D_{t}-1\right)D_{t}W_{t}/2, \end{array}$$

with $W_{t}|F_{t-1}∼P^{*}\left(\lambda_{t},K\right)$ and:

(3) $$\begin{array}{c} \lambda_{t}=\alpha_{0}+\alpha_{1}Y_{t-1}+\beta_{1}\lambda_{t-1}, \end{array}$$

where $\alpha_{0}>0,\alpha_{1}>0,\beta_{1}\geq 0,F_{t}$ is the available information up to time t, and $D_{t}$ satisfying Equation (2) is independent of $W_{t}$ . To achieve the stationary condition of Equation (3), Liu et al. [16] provide a sufficient condition as:

$$\begin{array}{c} \beta_{1}+K\left(1-\pi_{1}-\pi_{2}\right)\alpha_{1}/4<1. \end{array}$$

Note that Equation (3) is similar to the INGARCH(1,1) model of Weiß [19] for capturing the dynamic structure of $\lambda_{t}$ .

When $Y_{t}$ follows the ZOBPAR model, we can conduct the conditional probability of $Y_{t}$ as:

(4) $$\begin{array}{ccc} & & P\left(Y_{t}=k|F_{t-1}\right)=\pi_{1}I_{\left\{k=0\right\}}+\pi_{2}I_{\left\{k=1\right\}}+\left(1-\pi_{1}-\pi_{2}\right)\frac{\lambda_{t}^{k}/k!}{\sum_{i=0}^{K}\lambda_{t}^{i}/i!}, \end{array}$$

where k=0,1,…,K , and its conditional mean and variance are:

$$E\left(Y_{t}|F_{t-1}\right)=\pi_{2}+\left(1-\pi_{1}-\pi_{2}\right)\frac{g^{\prime }\left(\lambda_{t}\right)}{g(\lambda_{t})}\lambda_{t},$$

$$\begin{array}{cc} Var(Y_{t}|F_{t-1}) & =\pi_{2}\left(1-\pi_{2}\right)+\left(1-\pi_{1}-\pi_{2}\right)\frac{\left(1-2\pi_{2}\right)g^{\prime }\left(\lambda_{t}\right)}{g(\lambda_{t})}\lambda_{t}\\ \; & +\left(1-\pi_{1}-\pi_{2}\right)\left[\frac{g^{\prime \prime }\left(\lambda_{t}\right)}{g(\lambda_{t})}-\left(1-\pi_{1}-\pi_{2}\right){\left(\frac{g^{\prime }\left(\lambda_{t}\right)}{g(\lambda_{t})}\right)}^{2}\right]\lambda_{t}^{2}, \end{array}$$

where the function $g\left(\lambda_{t}\right)=\sum_{i=0}^{K}\lambda_{t}^{i}/i!$ , and $g^{\prime }\left(\lambda_{t}\right)$ and $g^{\prime \prime }\left(\lambda_{t}\right)$ are its first and second derivatives, respectively.

In addition to considering the effects of exogenous variables, we extend Equation (3) by incorporating these variables, denoted as $X_{j,t}$ . We then define the ZOBPAR model with exogenous variables (ZOBPARX) as:

(5) $$\begin{array}{c} \lambda_{t}=\alpha_{0}+\alpha_{1}Y_{t-1}+\beta_{1}\lambda_{t-1}+\sum_{j=1}^{S}\gamma_{j}X_{j,t-1}, \end{array}$$

where $\gamma_{j}$ is the parameter of the *j*th exogenous variable, and we restrict $\gamma_{j}>0$ to ensure non-negativeness of $\lambda_{t}$ . *S* is the number of exogenous variables.

## 3. Bayesian Inference and Forecasting

Let $\theta ={\left(\pi^{\prime },\alpha^{\prime }\right)}^{\prime }$ be the unknown parameter vector of the ZOBPAR model, where $\pi ={\left(\pi_{1},\pi_{2}\right)}^{\prime }$ , and $\alpha ={\left(\alpha_{0},\alpha_{1},\beta_{1}\right)}^{\prime }$ . Based on Equation (4), the log-likelihood function of $\left\{Y_{1},Y_{2},\cdots ,Y_{n}\right\}$ is:

$$\begin{array}{c} logL\left(\theta \right)=\sum_{t=2}^{n}l_{t}\left(\theta \right), \end{array}$$

and

$$\begin{array}{ccc} l_{t}\left(\theta \right) & = & I_{\left\{Y_{t}=0\right\}}\log(\pi_{1}+\left(1-\pi_{1}-\pi_{2}\right)/g\left(\lambda_{t}\right)\\ & + & I_{\left\{Y_{t}=1\right\}}\log(\pi_{2}+\left(1-\pi_{1}-\pi_{2}\right)\lambda_{t}/g\left(\lambda_{t}\right)\\ & + & I_{\left\{Y_{t}\geq 2\right\}}\log(\left(1-\pi_{1}-\pi_{2}\right)\lambda_{t}^{Y_{t}}/\left(Y_{t}!g\left(\lambda_{t}\right)\right), \end{array}$$

where $\lambda_{t}$ is defined by Equation (3) or (5), and $g\left(\lambda_{t}\right)=\sum_{i=0}^{K}\lambda_{t}^{i}/i!$ .

Using Bayes’ rule, we multiply the likelihood and the priors to give the conditional posterior probability as follows:

$$\begin{array}{c} p\left(\theta_{l}|Y,X,\theta_{\neq l}\right)\propto L\left(Y|X,\theta \right)\times p\left(\theta_{l}\right), \end{array}$$

where $Y={\left(Y_{1},\cdots ,Y_{n}\right)}^{\prime }$ , and $X={\left(X_{1},\cdots ,X_{n}\right)}^{\prime }$ . Here, $\theta_{l}$ denotes each parameter in θ , $p(\theta_{l})$ is the prior density of $\theta_{l}$ , and $\theta_{\neq l}$ is the vector of all model parameters except for $\theta_{l}$ . We choose noninformative priors for all parameters and restrict the parameters to satisfy the specified region. $p\left(\pi \right)=I\left(A_{1}\right)$ , and $p\left(\alpha \right)=I\left(A_{2}\right)$ , where $A_{1}=\left\{\pi_{1}\geq 0,\pi_{2}\geq 0\;and\;\pi_{1}+\pi_{2}<1\right\}$ and $A_{2}=\left\{\alpha_{0}>0,\alpha_{1}>0,\beta_{1}\geq 0\;and\;\beta_{1}+K\left(1-\pi_{1}-\pi_{2}\right)\alpha_{1}/4<1\right\}$ .

### 3.1. MCMC Sampling Schemes

We designed an MCMC sampling scheme to obtain the posterior estimates of θ for the ZOBPAR model using an adaptive Metropolis-Hastings (MH) algorithm by Chen and So [20]. This approach combines the random walk Metropolis (RW-M) algorithm and the independent kernel MH (IK-MH) algorithm, with a total number of iterations *N* and burn-in iterations *M*. The steps of the MCMC sampling scheme are as follows.

Step 1: Give initial values of $\theta^{\left[0\right]}={\left(\pi_{1}^{\left[0\right]},\pi_{2}^{\left[0\right]},\alpha_{0}^{\left[0\right]},\alpha_{1}^{\left[0\right]},\beta_{1}^{\left[0\right]}\right)}^{\prime }$ .
Step 2: At the *i*th iteration, update $\pi^{\left[i\right]}={\left(\pi_{1}^{\left[i\right]},\pi_{2}^{\left[i\right]}\right)}^{\prime }$ by the MH algorithm conditional on $\alpha^{\left[i-1\right]}$ . If i≤M , then the RW-M algorithm is used; otherwise, the IK-MH algorithm is employed.
Step 3: Similarly, at the *i*th iteration, $\alpha^{\left[i\right]}={\left(\alpha_{0}^{\left[i\right]},\alpha_{1}^{\left[i\right]},\beta_{1}^{\left[i\right]}\right)}^{\prime }$ is updated by the MH algorithm conditional on $\pi^{\left[i\right]}$ . If i≤M , then the RW-M algorithm is used; otherwise, the IK-MH algorithm is employed.
Step 4: Repeat Step 2 and Step 3 until the number of iterations equals *N*.

The procedures for the RW-M and IK-MH algorithms for α run as follows.

(i) The steps of RW-M for α from i=1,…,M are as follows.
  Step 1:
$\alpha^{*}=\alpha^{\left[i-1\right]}+\epsilon$ , where ϵ∼N(0,cI) , *I* is identity matrix, and $\alpha^{\left[i-1\right]}$ is the estimate of α at the (*i*− 1)th iteration.
  Step 2: Accept $\alpha^{*}$ as $\alpha^{\left[i\right]}$ with acceptance probability:
$$Pr=\min\left\{\begin{array}{c} 1,\frac{p(\alpha^{*})}{p(\alpha^{\left[i-1\right]})} \end{array}\right\}>u,\mathrm{where}\;u∼U\left(0,1\right),$$
    where p(α) is the conditional posterior distribution of α . If u<Pr , set $\alpha^{\left[i\right]}=\alpha^{*}$ ; otherwise, set $\alpha^{\left[i\right]}=\alpha^{\left[i-1\right]}$ .
  Step 3: Repeat Step 1 and Step 2 for each MCMC iteration during the burn-in iterations.
  Note that the stepsize *c* in Step 1 controls the acceptance rate for α .
  According to Gelman et al. [21], a suitable value of the acceptance rate for good convergence is between 25% and 50% .
(ii) The steps of IK-MH for α from i=M+1,…,N are as follows:
  Step 1:
$\alpha^{*}=\mu_{\alpha }+\epsilon$ , where $\epsilon ∼N(0,\Omega_{\alpha })$ , with $\mu_{\alpha }$ and $\Omega_{\alpha }$ as the sample mean and sample variance of the estimates of α from the burn-in samples.
  Step 2: Update $\alpha^{*}$ as $\alpha^{\left[i\right]}$ with acceptance probability:
$$Pr=\min\left\{\begin{array}{c} 1,\frac{p\left(\alpha^{*}\right)g\left(\alpha^{\left[i-1\right]}\right)}{p\left(\alpha^{\left[i-1\right]}\right)g\left(\alpha^{*}\right)} \end{array}\right\}>u,\mathrm{where}\;u∼U\left(0,1\right),$$
    where *g* is a Gaussian proposal density with mean $\mu_{\alpha }$ and variance $\Omega_{\alpha }$ . If u<Pr , set $\alpha^{\left[i\right]}=\alpha^{*}$ ; otherwise, set $\alpha^{\left[i\right]}=\alpha^{\left[i-1\right]}$ .
  Step 3: Repeat Step 1 and Step 2 until the total number of iterations is *N*.

The RW-M and IK-MH procedures for π are similar to the procedures of α .

### 3.2. Bayesian Forecasting

In the empirical illustration, we conduct one-step-ahead forecasting to predict ${\hat{Y}}_{t+1}$ . The Bayesian forecasting procedure runs as follows (for example, K=4 ):

Step 1: Obtain the posterior means of parameters $\hat{\theta }={\left(\hat{\pi },\hat{\alpha }\right)}^{\prime }$ , where $\hat{\pi }={\left({\hat{\pi }}_{1},{\hat{\pi }}_{2}\right)}^{\prime }$ and $\hat{\alpha }={\left({\hat{\alpha }}_{0},{\hat{\alpha }}_{1},{\hat{\beta }}_{1}\right)}^{\prime }$ ,
$${\hat{\pi }}_{j}=\frac{\sum_{i=M}^{N}\pi_{j}^{\left[i\right]}}{N-M},\;j=1,2,\;\mathrm{and}\;{\hat{\alpha }}_{j}=\frac{\sum_{i=M}^{N}\alpha_{j}^{\left[i\right]}}{N-M},\;j=0,1,2,\;\left(\mathrm{where}\;{\hat{\alpha }}_{2}={\hat{\beta }}_{1}\right),$$
  and then put $\hat{\alpha }={\left({\hat{\alpha }}_{0},{\hat{\alpha }}_{1},{\hat{\beta }}_{1}\right)}^{\prime }$ into Equation (3) to calculate ${\hat{\lambda }}_{t+1}={\hat{\alpha }}_{0}+{\hat{\alpha }}_{1}Y_{t}+{\hat{\beta }}_{1}\lambda_{t}.$
Step 2: Compute the conditional probability of:
$$P\left(Y_{t+1}=k|\theta ,F_{t}\right)={\hat{\pi }}_{1}I_{\left\{k=0\right\}}+{\hat{\pi }}_{2}I_{\left\{k=1\right\}}+\left(1-{\hat{\pi }}_{1}-{\hat{\pi }}_{2}\right)\frac{{\hat{\lambda }}_{t+1}^{k}/k!}{\sum_{i=0}^{K}{\hat{\lambda }}_{t+1}^{i}/i!},$$
  where k=0,1,2,3,4 by $\hat{\pi }={\left({\hat{\pi }}_{1},{\hat{\pi }}_{2}\right)}^{\prime }$ and ${\hat{\lambda }}_{t+1}$ .
Step 3: Generate a random number u∼U(0,1) .
  (1) If $u\leq P(Y_{t+1}=0)$ , then ${\hat{Y}}_{t+1}=0$ ; else, move to the next statement.
  (2) If $P\left(Y_{t+1}=0\right)<u\leq P\left(Y_{t+1}\leq 1\right)$ , then ${\hat{Y}}_{t+1}=1$ ; else, move to the next statement.
  (3) If $P\left(Y_{t+1}\leq 1\right)<u\leq P\left(Y_{t+1}\leq 2\right)$ , then ${\hat{Y}}_{t+1}=2$ ; else, move to the next statement.
  (4) If $P\left(Y_{t+1}\leq 2\right)<u\leq P\left(Y_{t+1}\leq 3\right)$ , then ${\hat{Y}}_{t+1}=3$ ; else, move to the next statement.
  (5)
${\hat{Y}}_{t+1}=4$ .

We employ this procedure to generate one-step-ahead forecasts for bounded ordinal time series data.

## 4. Simulation Study

In this section we conduct a simulation study to examine the established Bayesian MCMC method. There are two models: Model 1 is a ZOBPAR model, and Model 2 is a ZOBPARX model, specified as follows to generate simulated data with sample size n=500 and n=1000 :

**Model 1:**

$$\begin{array}{ccc} & & \lambda_{t}=0.02+0.7y_{t-1}+0.2\lambda_{t-1},\\ & & P\left(Y_{t}=k|F_{t-1}\right)=0.01I_{\left\{k=0\right\}}+0.3I_{\left\{k=1\right\}}+0.69\frac{\lambda_{t}^{k}/k!}{\sum_{i=0}^{4}\lambda_{t}^{i}/i!},\;k=0,1,\ldots ,4,\\ & & \pi =\left(\pi_{1},\pi_{2}\right)=\left(0.01,0.3\right);\;\alpha =\left(\alpha_{0},\alpha_{1},\beta_{1}\right)=\left(0.02,0.7,0.2\right). \end{array}$$

**Model 2:**

$$\begin{array}{ccc} & & \lambda_{t}=0.02+0.7y_{t-1}+0.2\lambda_{t-1}+0.3X_{t-1},\\ & & P\left(Y_{t}=k|F_{t-1}\right)=0.01I_{\left\{k=0\right\}}+0.3I_{\left\{k=1\right\}}+0.69\frac{\lambda_{t}^{k}/k!}{\sum_{i=0}^{4}\lambda_{t}^{i}/i!},\;k=0,1,\ldots ,4,\\ & & \pi =\left(\pi_{1},\pi_{2}\right)=\left(0.01,0.3\right);\;\alpha =\left(\alpha_{0},\alpha_{1},\beta_{1},\gamma_{1}\right)=\left(0.02,0.7,0.2,0.3\right). \end{array}$$

We generate $X_{t}$ in Model 2 from a Gamma distribution with $X_{t}∼G\left(2,2\right)$ . Both models follow a bounded Poisson distribution with bound K=4 .

A computational issue arises with a slow convergence rate of parameter estimates, when we simultaneously estimate a set of unknown parameters. To implement the estimation of model parameters, we use the Bayesian method with a designed MCMC sampling. We employ two sampling mechanisms to speed up the convergence of MCMC sampling. First, we use an adaptive MCMC sampling method, which combines the RW-M algorithm and the IK-MH algorithm, as mentioned in the Section 3.1. Second, due to autocorrelation, we choose every five samplers as a thinning chain in MCMC outputs. The total number of MCMC iterations is 20,000, which includes a burn-in period of 8000 iterations. Based on R codes, the computational times for the ZOBPAR model with sample sizes n=500 and n=1000 are 159 and 309 s, respectively. For the ZOBPARX model, the CPU times are approximately 241 and 464 s. Parameter estimations are efficiently completed in under eight minutes, and all computations are performed on a computer equipped with an i7-11700 CPU and 64 GB of RAM.

**Figure 1:**
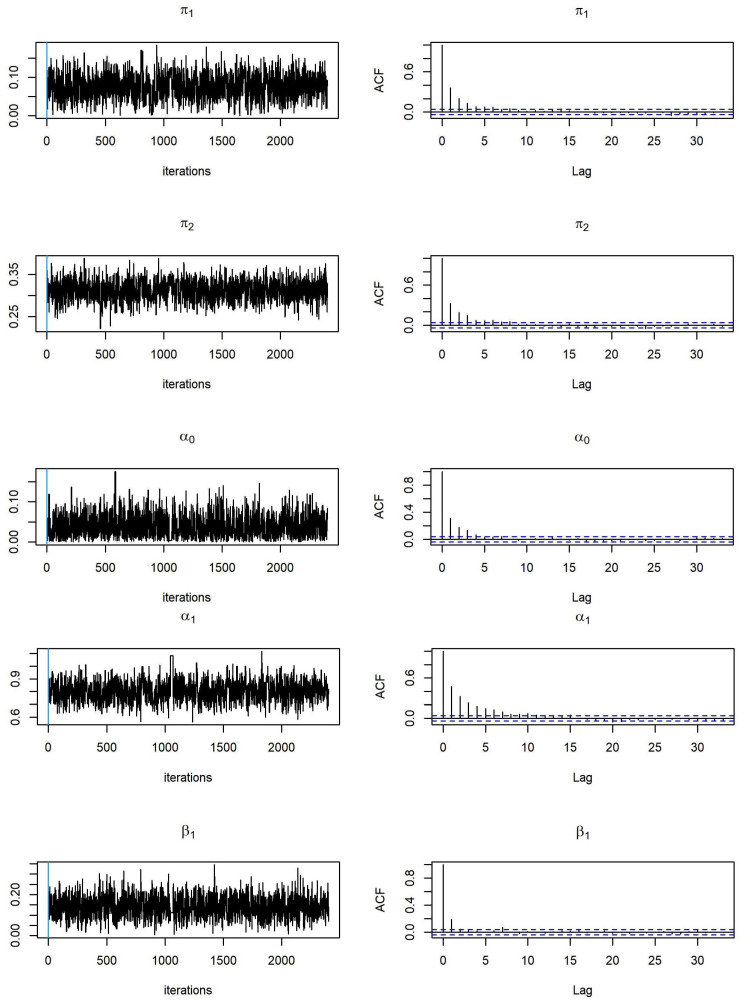
Trace plots and ACF plots of parameter estimates related to the ZOBPAR model (Model 1).

**Figure 2:**
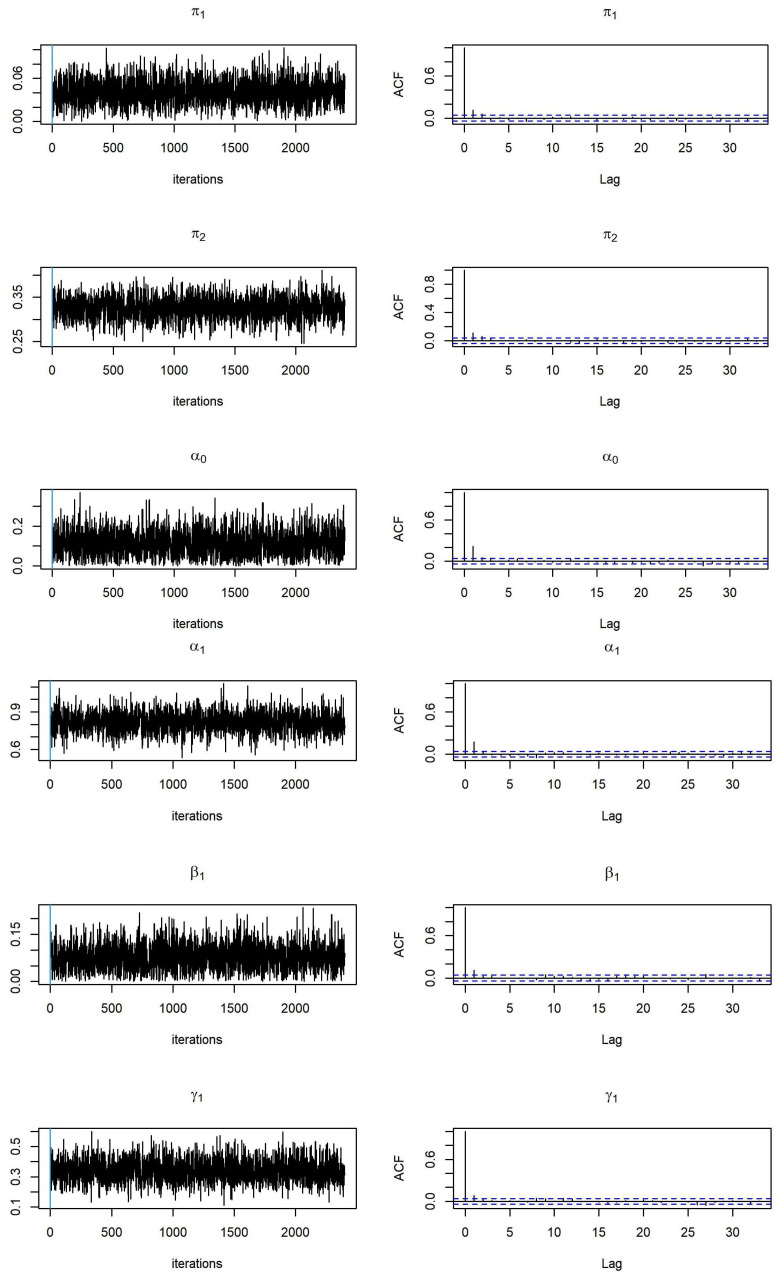
Trace plots and ACF plots of parameter estimates related to the ZOBPARX model (Model 2).

To examine the convergence of MCMC, we monitor the trace and autocorrelation function (ACF) plots of MCMC samplers during the after burn-in iterations. We provide trace plots and ACF plots for MCMC samples based on simulated data from Model 1 and Model 2 with sample size n=1000 (see Figure 1 and Figure 2). We expect that the trace plots of all parameters randomly vary between a reasonable constant range, and that the ACF plots present no autocorrelation observed in the MCMC samplers. This demonstrates that we have converged MCMC samplers, and that the parameter estimates are reliable.

The results in Table 1 reveal that the averages of posterior means based on 100 replications are close to the true values, and 95% Bayesian credible intervals ( ${℘}_{2.5}$ and ${℘}_{97.5}$ ) can accurately cover the corresponding true values. This confirms that the designed MCMC method provides accurate parameter estimates. To examine the consistency of parameter estimates, we offer the results of parameter estimates for different sample sizes n=500 and 1000. The results indicate that the proposed Bayesian method presents accurate parameter estimates with small standard deviations as the sample size increases.

The results of parameter estimates for the ZOBPARX model (Model 2) are presented in Figure 2 and Table 2. All MCMC samples converge, and the posterior means are close to the true values. Again, all parameter estimates are close to the true values, while the posterior standard deviations are small as the sample size increases.

## 5. Empirical Application

In order to demonstrate our proposed method, we investigate daily AQI levels from the weather stations of Pingtung, Miaoli, and Zuoying in Taiwan. We collect daily AQI levels for each station from 30 December 2016 to 31 January 2020 for a total of 1129 observations. To evaluate the forecasting performance, we separate the whole sample period into two periods: the training period with 764 observations for in-sample model estimation and the testing period with 365 days for out-of-sample forecasts. By a rolling window approach, we conduct one-step ahead forecasts for 365 days and evaluate the forecast performance by computing the accuracy of AQI level forecasts.

Precipitation can effectively reduce particulate matter concentrations (PM_10_ and PM_2.5_) in the air. When it rains, these particles are captured by raindrops and carried to the ground. This process can lead to a significant improvement in AQI, particularly in reducing particulate pollution. We thus treat daily accumulated precipitation (PRE) and the winter dummy variable as exogenous variables and consider different combinations of ZOBPARX models to study the effects of exogenous variables. We define daily accumulated PRE as an exogenous variable $X_{t}$ , which negatively correlates with the AQI level. We propose two distinct transformations to fulfil the coefficient constraint ( $\gamma_{j}>0$ ) and to address the negative correlation between yesterday’s PRE and today’s AQI level. The first transformation involves taking the reciprocal of PRE (TF1_PRE), set as $X_{1,t}$ , and the second involves computing the exponential of the negative PRE (TF2_PRE), designated as $X_{2,t}$ .

$$\begin{array}{ccc} & & TF1\_PRE:\;X_{1,t}=1/\left(X_{t}+1\right)\;\mathrm{and}\\ & & TF2\_PRE:\;X_{2,t}=\exp\left(-X_{t}\right). \end{array}$$

These transformations of $X_{t}$ can ensure having positive estimates of the coefficients in the ZOBPARX model. To investigate the effect of seasonality, we consider the exogenous variables of the winter dummy variable and month dummy variables. For the winter dummy variable, we define $S_{t}$ as:

$$S_{t}=\left\{\begin{array}{cc} 1, & \mathrm{from}\;\mathrm{October}\;\mathrm{to}\;\mathrm{March},\\ 0, & \mathrm{otherwise}. \end{array}\right.$$

According to the definitions of exogenous variables, we consider following ZOBPARX models with different combinations of exogenous variables for three datasets.

**ZOBPARX 1:** (exogenous variable: TF1_PRE)
$$\begin{array}{cc} \lambda_{t} & =\alpha_{0}+\alpha_{1}Y_{t-1}+\beta_{1}\lambda_{t-1}+\gamma_{1}X_{1,t-1}. \end{array}$$
**ZOBPARX 2:** (exogenous variable: TF2_PRE)
$$\begin{array}{cc} \lambda_{t} & =\alpha_{0}+\alpha_{1}Y_{t-1}+\beta_{1}\lambda_{t-1}+\gamma_{1}X_{2,t-1}. \end{array}$$
**ZOBPARX 3:** (exogenous variables: TF1_PRE and winter dummy)
$$\begin{array}{cc} \lambda_{t} & =\alpha_{0}+\alpha_{1}Y_{t-1}+\beta_{1}\lambda_{t-1}+\gamma_{1}X_{1,t-1}+\gamma_{2}S_{t}. \end{array}$$
**ZOBPARX 4:** (exogenous variables: TF2_PRE and winter dummy)
$$\begin{array}{cc} \lambda_{t} & =\alpha_{0}+\alpha_{1}Y_{t-1}+\beta_{1}\lambda_{t-1}+\gamma_{1}X_{2,t-1}+\gamma_{2}S_{t}. \end{array}$$

In the process of data collection, we faced problems of missing observations in AQI and weather covariate. To avoid loss of information, we adopt the k-nearest neighbors (knn) algorithm by Cover and Hart [22] to impute the missing values, which takes the same approach as Chen and Chiu [15].

Chiu [23] conducted an experiment on a selected period of the AQI time series without missing values from three randomly chosen sites. Chiu [23] then introduced missing values at every 10th datapoint and imputed these using the KNN-imputation method from the R package “DMwR” [24]. This process compares different *h* values for AQI prediction, using some variables to minimize the mean absolute error (MAE) and root mean squared error (RMSE). The results suggest that employing four days ( h=4 ) with data on rainfall, temperature, wind direction, PM_2.5_, and seasonal dummy variables, which are closest to the day with missing AQI, and then taking the weighted average of the AQI values from these four days, serves as an effective imputation for the missing AQI. Following the same line, we impute missing values and refer to the results of Chiu [23] and Chen and Chiu [15] to set h=4 for the imputation of missing values in PRE and AQI, as shown below.

(1) PRE with missing values: If the PRE value is missing at day *t*, then we pick the valid data of the nearest station to impute;
(2) AQI with missing values: In the knn algorithm, we set h=4 to impute the missing AQI values by the corresponding weather data in the nearest four days concerning PRE, daily average temperature, daily wind direction, PM_2.5_, and seasonal dummy value closest to the day with missing AQI; the imputation of a missing AQI value is the weighted average of the AQI values of these four days. The weights decrease as the distances to their neighbor increase, and we use a Gaussian kernel function to take the weights from their distances. For more details, one can refer to Torgo [24].

**Figure 3:**
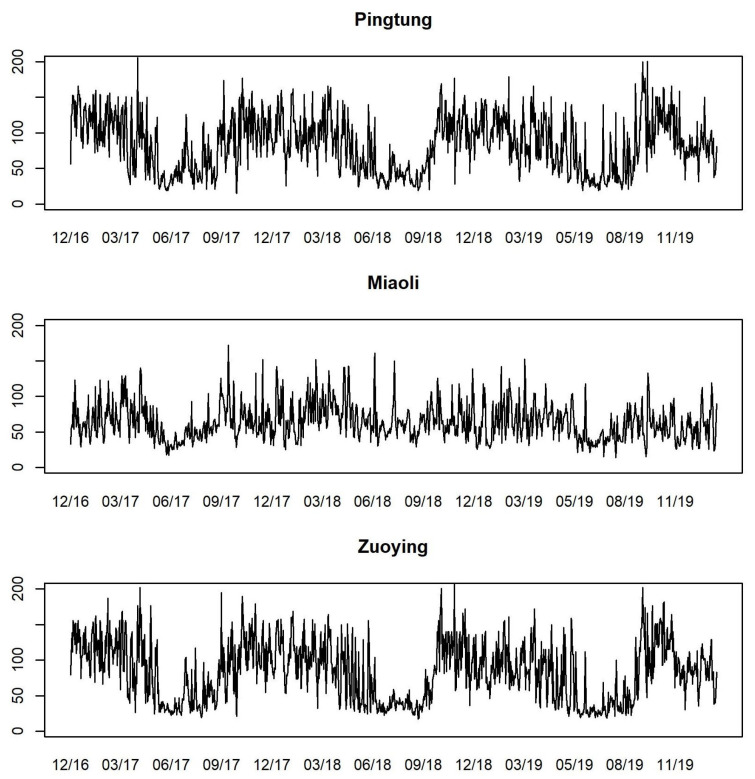
Time series plots of daily AQI values for Pingtung, Miaoli, and Zuoying from 30 December 2016 to 31 January 2020.

**Figure 4:**
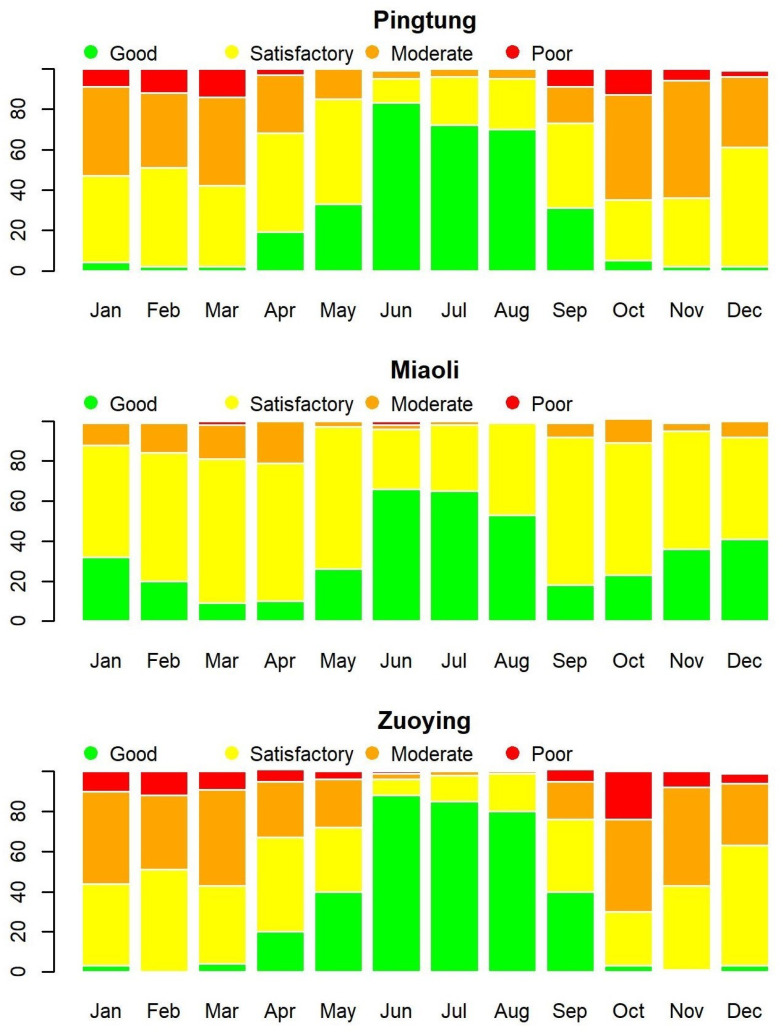
Monthly AQI levels for Pingtung, Miaoli, and Zuoying from 30 December 2016 to 31 January 2020.

Following the U.S. EPA’s classification of AQI levels, we classify AQI values into four levels, each represented by different colors for visual identification, as in Table 3. Figure 3 presents the time series plots of daily AQI values from 30 December 2016 to 31 January 2020 for Pingtung, Miaoli, and Zuoying. Observing these time series plots, the changes of AQI values in Pingtung and Zuoying are more volatile than in Miaoli, and the AQI values are low from May to September for each year at each station. A large proportion of AQI values under 100 at the three stations distinctly demonstrates the phenomenon of zero-one inflation in the data concerning AQI levels. Figure 4 plots the proportions of AQI levels by stacked bar charts for each month and each station. It is obvious that the levels of AQI are quite different among months, while the period from June to August has better air quality, with large proportions of 0 and 1 levels of AQI.

**Figure 5:**
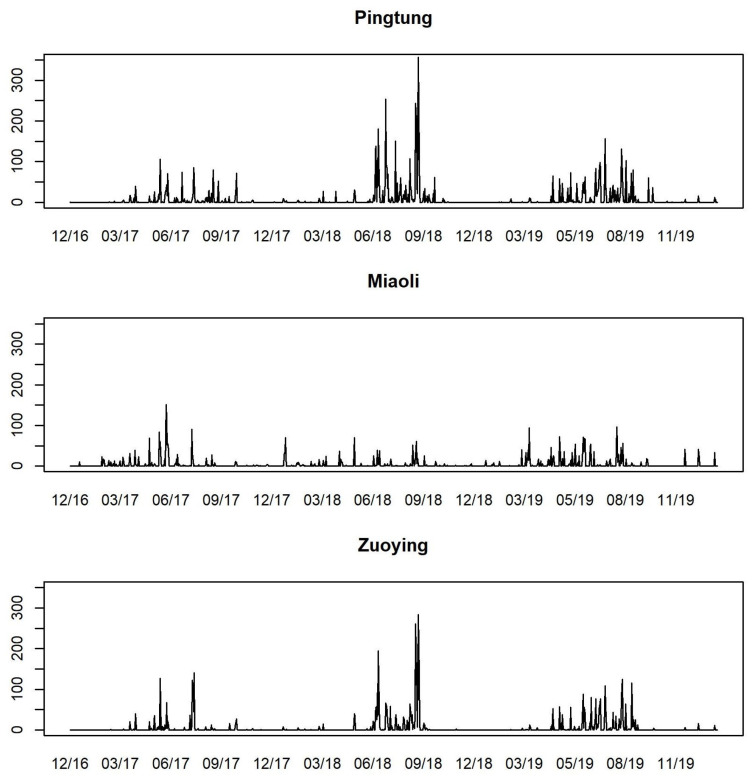
Time series plots of daily PRE values for Pingtung, Miaoli, and Zuoying from 30 December 2016 to 31 January 2020.

The time series plots of daily PRE for the three stations, presented as the exogenous variable PRE in Figure 5, indicate that the rainy season occurs periodically from May to September. The highest daily cumulative PRE typically occurs during July and August. We provide the summary statistics of AQI and PRE for the three stations in Table 4. The means and standard deviations of AQI of Pingtung and Zuoying are both larger than their values for Miaoli. This is consistent with the findings in Figure 3. The maximum values of AQI of Pingtung and Zuoying both exceed 200, which is at the level of “poor”. Similar to the PRE values, Pingtung and Zuoying have larger mean values and standard deviations of PRE than Miaoli. We observe that the weather is relatively unstable in Pingtung and Zuoying.

We employ MCMC methods for parameter estimation and one-step-ahead AQI forecasts during the out-of-sample period for each dataset. To evaluate the performance of our proposed method in forecasting AQI levels, we fit a ZOBPAR model and four ZOBPARX models, using each to perform one-step-ahead forecasts for 365 days through the rolling window method. To the best of our knowledge, apart from the ZOBPAR model, no other model can investigate zero-one inflation with a bounded Poisson distribution. Therefore, we consider a ZOBPAR model and four ZOBPARX models for the comparative analysis. Table 5 presents two evaluation metrics—accuracy and penalty—used to assess the AQI level forecasts over a period of 365 days for each dataset, as predicted by these five different models.

Categories 0 and 1 of AQI levels both represent satisfactory air quality and have no effect on human health, whereas other categories are harmful to human health. We treat all categories split into two levels for calculating the accuracy of AQI-level forecasts. To evaluate the effectiveness of model prediction, we propose a penalty mechanism that reflects a numerical ‘cost’ or ‘penalty’ for each type of misclassification in our categorical data. The scores of penalty in Table 5 are the sum of daily penalties during the forecasting period. The lower the penalty score, the better the model.

We assign a numerical ‘cost’ or ‘penalty’ to each type of misclassification in our categorical data, adopting a concept akin to weighted absolute error. This approach involves defining a weight or cost for each type of error (e.g., predicting Category A when the actual category is B) and then calculating an overall score based on these weights. If there are differences between actual AQI levels and predicted AQI levels, denoted as $w_{i}\left|Y_{i}-{\hat{Y}}_{i}\right|$ , are −1, −2, 1, or 2, then we design a penalty mechanism with corresponding weights of 1, 2, 4, and 8, respectively. The aim of this method is to ensure that the weights accurately reflect the relative importance of each category, with the underestimated category receiving a higher penalty than the overestimated one, and to accurately represent the cost of each type of error in our specific context.

For the Pingtung site, the ZOBPARX 1 model shows the highest accuracy (67.4%), indicating it most frequently predicts the AQI levels correctly. ZOBPARX 1 also leads with a lower penalty score than the other models. For the Miaoli site, the ZOBPARX 3 model has the highest accuracy (87.9%) and the lowest penalty. For the Zuoying site, the ZOBPARX 3 model shows the highest accuracy (69.9%) but the ZOBPAR model provides the lowest penalty. The ZOBPARX 2 model performs as the second-best model, provideing reasonable accuracy and a low penalty.

It appears that the models generally perform well in forecasting for Miaoli’s air quality. In contrast, these models exhibit lower accuracy in Pingtung and Zuoying. In summary, different models excel in different locations, highlighting the importance of selecting location-specific models for accurate AQI forecasting.

The Environmental Protection Bureau of the Pingtung County government states that the county’s geographic location at the southernmost end, often positioned downwind of prevailing winds, contributes to its air pollution issues. The weaker wind speeds in winter, fewer days of rainfall, and poor atmospheric dispersion conditions, compounded by the region’s topography that can create localized eddy currents, are all factors that lead to higher concentrations of air pollutants in Pingtung County during the autumn and winter seasons, often exceeding standard levels [25]. We need to tailor models for local geographical and environmental characteristics to improve forecast accuracy for specific regions like Pingtung.

For the three datasets, we present the posterior estimates of parameters for the best performing models in Table 6 and also provide convergence diagnostic checking, the convergence diagnostic (CD) test, and inefficiency factors (Ineff.) to demonstrate converged parameter estimates. The CD test introduced by Geweke [26] has a *p*-value greater than 0.05, and the Ineff. of Chib [27] has a small value that is far less than MCMC iterations; both reveal that the chain of MCMC samples converges. The last two columns of Table 6 present the *p*-values of CD tests and the Ineff. values. All *p*-values of CD tests are greater than 0.05, and all Ineff. values are far smaller than MCMC iterations. This means that all parameter estimates converge and are reliable for making inferences.

To check the adequacy of the fitted model, we compute standardized Pearson’s residuals by Jung et al. [28] as follows:

$$\begin{array}{c} Z_{t}=\frac{y_{t}-E\left(Y_{t}|F_{t-1}\right)}{\sqrt{Var(Y_{t}|F_{t-1})}},t=1,\ldots ,n. \end{array}$$

For Zuoying, in Table 6 we observe that the posterior estimate of $\alpha_{1}$ in the ZOBPARX 2 model is larger than $\alpha_{0}$ and $\beta_{1}$ . This indicates that the AQI level of the previous day has a significant effect on the current day’s mean AQI level. The estimates of the probabilities $\pi_{1}$ and $\pi_{2}$ show that $\pi_{2}$ is larger than $\pi_{1}$ in the case of Zuoying. Focusing on the parameters of exogenous variables, the parameter $\gamma_{1}$ for $X_{2,t}$ in the ZOBPARX 2 model for Pingtung has an estimate of 0.0457.

For Pingtung, the ZOBPARX 1 model produces more accurate predictions among the competing models. Bayesian parameter estimations for the ZOBPARX 1 model are presented in Table 6. We also observe that the posterior estimate of $\alpha_{1}$ has a larger magnitude than that of $\alpha_{0}$ and $\beta_{1}$ , and again the estimate of $\pi_{2}$ is greater than $\pi_{1}$ at this site.

For Miaoli, the ZOBPARX 3 model is the best-performing model with the highest accuracy among the competing models. The parameter estimation results for the ZOBPARX 3 model are presented in Table 6. The posterior estimate of $\alpha_{1}$ is again larger than both $\alpha_{0}$ and $\beta_{1}$ , and the estimates of $\pi_{1}$ and $\pi_{2}$ reveal that $\pi_{2}$ is larger than $\pi_{1}$ in the Miaoli dataset. When examining the parameters of exogenous variables, the parameters $\gamma_{1}$ and $\gamma_{2}$ for $X_{1,t}$ and $S_{t}$ in the ZOBPARX 3 model for Miaoli demonstrate a much smaller magnitude on AQI levels, with estimates of 0.0200 and 0.0478, respectively.

These findings underscore the significance of $\alpha_{1}$ in AQI forecasting models for each site, emphasizing the crucial influence of the previous day’s AQI levels. Consistently across the three sites, the estimate remains $\pi_{2}>\pi_{1}$ . Furthermore, PRE or the winter dummy variable plays an important role in the forecasts.

**Figure 6:**
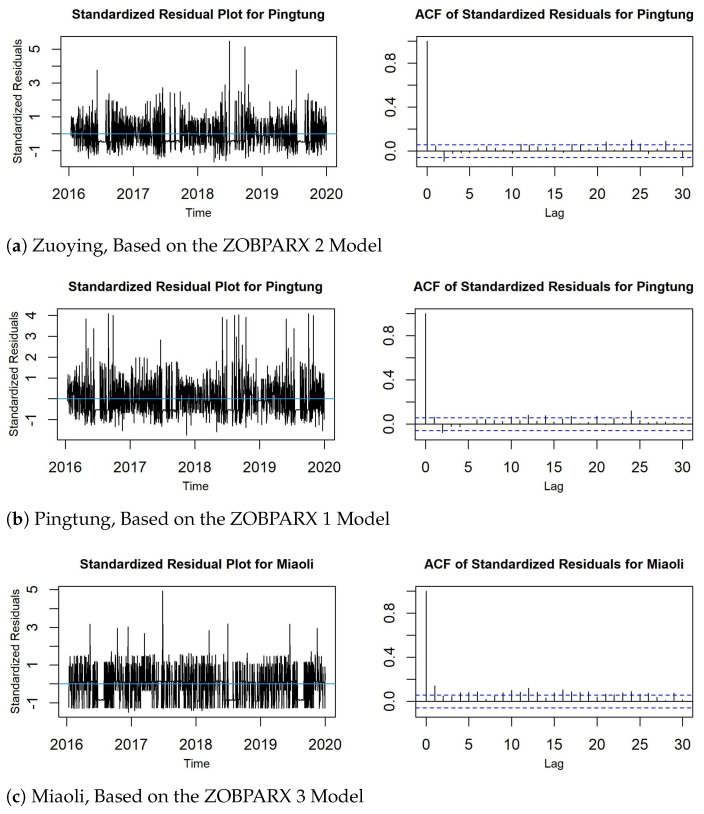
Time plots and ACF plots of standardized Pearson’s residuals for: (**a**) Zuoying, (**b**) Pingtung, and (**c**) Miaoli.

Figure 6 displays the time plots and ACF plots of the standardized Pearson’s residuals for the three sites, derived using the most accurate forecasting models. The diagnostic checking results suggest that the proposed models adequately capture the changes in AQI levels at these sites.

To gain a detailed understanding of the performances of AQI level forecasts in each dataset, we compute the proportions of forecasted AQI levels $\left(\hat{Y_{t}}\right)$ and compare them with the actual proportions of AQI levels observed during the 365-day out-of-sample forecasting period, as shown in Table 7. Focusing on the results of Miaoli, the best model obtained in Table 5 provides accurate results on AQI level forecasts, with the proportions of forecasted levels $\left(\hat{Y_{t}}\right)$ close to the true proportions. In Pingtung, due to local geographical and environmental conditions, even the best forecasting model does not perform well in terms of the proportions of forecasted AQI levels.

## 6. Conclusions

This paper presents the issues of modeling and forecasting bounded ordinal time series data with a special focus on AQI levels. The data are bounded and predominantly concentrated on a few categories, such as states 0 and 1, which occur with high probabilities. We demonstrate ZOBPAR models, both with and without exogenous variables, in order to capture the dynamic changes in such ordinal time-series data. We propose a Bayesian inference method that utilizes an effective MCMC sampling mechanism. This method estimates model parameters and forecasts one-step-ahead AQI levels using a rolling window approach. To check the convergency of MCMC samplers, we monitor the trace and ACF plots by visual inspection and compute Geweke’s convergence diagnostic.

Simulation studies demonstrate that the proposed method provides reliable model parameter estimates, and the posterior means of these estimates are robustly close to the actual values as the sample size increases. For the empirical study, we investigate three datasets of daily AQI levels from Pingtung, Zuoying, and Miaoli stations in Taiwan. Apart from Pingtung County, the prediction outcomes demonstrate that the proposed method effectively forecasts AQI levels during the testing period. This is evident from the alignment of predicted AQI proportions with the actual proportions, especially notable at the Miaoli station. To enhance forecasting accuracy for Pingtung, it is essential to customize models that fit its unique geographical and environmental characteristics.

Aside from using parametric models, there are some machine learning methods for forecasting bounded ordinal time series data, including tree-based models, neural networks, and support vector machines. We propose the consideration of model-averaging forecasts for ordinal and bounded time series data. The advantage of averaging over multiple models is the reduced risk of relying on a single, potentially inappropriate model, which can also enhance accuracy by balancing out biases inherent in individual models. We plan to explore this aspect in our future work.

## Figures and Tables

**Table 1:** Parameter estimates of the ZOBPAR model (Model 1) based on 100 replications.

Parameter	True	Mean	Median	Std	${℘}_{2.5}$	${℘}_{97.5}$
n=500						
$\pi_{1}$	0.01	0.0559	0.0497	0.0379	0.0035	0.1437
$\pi_{2}$	0.30	0.3024	0.3029	0.0334	0.2359	0.3667
$\alpha_{0}$	0.02	0.0573	0.0498	0.0400	0.0039	0.1524
$\alpha_{1}$	0.70	0.7450	0.7378	0.1047	0.5596	0.9759
$\beta_{1}$	0.20	0.1670	0.1634	0.0712	0.0413	0.3153
n=1000						
$\pi_{1}$	0.01	0.0404	0.0360	0.0270	0.0026	0.1032
$\pi_{2}$	0.30	0.2992	0.2995	0.0242	0.2512	0.3460
$\alpha_{0}$	0.02	0.0434	0.0384	0.0286	0.0043	0.1111
$\alpha_{1}$	0.70	0.7427	0.7389	0.0732	0.6106	0.8998
$\beta_{1}$	0.20	0.1747	0.1737	0.0517	0.0774	0.2786

**Table 2:** Parameter estimates of the ZOBPARX model (Model 2) based on 100 replications.

Parameter	True	Mean	Median	Std	${℘}_{2.5}$	${℘}_{97.5}$
n=500						
$\pi_{1}$	0.01	0.0382	0.0354	0.0224	0.0044	0.0881
$\pi_{2}$	0.30	0.3143	0.3143	0.0351	0.2454	0.3829
$\alpha_{0}$	0.02	0.1310	0.1179	0.0860	0.0091	0.3286
$\alpha_{1}$	0.70	0.7453	0.7417	0.1126	0.5351	0.9773
$\beta_{1}$	0.20	0.1505	0.1462	0.0728	0.0258	0.3029
$\gamma_{1}$	0.30	0.3073	0.2985	0.1109	0.1148	0.5474
n=1000						
$\pi_{1}$	0.01	0.0266	0.0247	0.0154	0.003	0.0608
$\pi_{2}$	0.30	0.3112	0.3112	0.0250	0.2622	0.3602
$\alpha_{0}$	0.02	0.0973	0.0885	0.0621	0.0069	0.2379
$\alpha_{1}$	0.70	0.7397	0.7380	0.0775	0.5926	0.8965
$\beta_{1}$	0.20	0.1508	0.1495	0.0563	0.0458	0.2643
$\gamma_{1}$	0.30	0.2965	0.2928	0.0734	0.1627	0.4496

**Table 3:** Classification of AQI levels.

Category	Value	Color	AQI Levels
0	0–50	Green	Good
1	51–100	Yellow	Satisfactory
2	101–150	Orange	Moderately
3	151 or more	Red	Poor

**Table 4:** Summary statistics of daily AQI and PRE values.

Station	Mean	Std	Min	Max
AQI				
Pingtung	83.9	39.6	15.0	206.0
Miaoli	63.9	25.5	14.0	172.0
Zuoying	83.2	42.2	17.0	210.0
PRE				
Pingtung	6.7	24.6	0.0	356.5
Miaoli	3.8	12.4	0.0	151.0
Zuoying	5.1	20.7	0.0	283.5

**Table 5:** Accuracy percentages and penalty scores of AQI level forecasts for Pingtung, Miaoli, and Zuoying from the considered models.

	Pingtung			Miaoli			Zuoying		
**Model**	**Accuracy (%)**	**Penalty**		**Accuracy (%)**	**Penalty**		**Accuracy (%)**	**Penalty**	
ZOBPAR	61.6	398		85.2	123		65.8	366	
ZOBPARX 1	67.4	377		84.9	135		68.5	395	
ZOBPARX 2	67.1	416		86.8	123		69.3	367	
ZOBPARX 3	65.5	411		87.9	111		69.9	391	
ZOBPARX 4	64.4	448		85.8	119		68.8	388	

**Table 6:** Parameter estimation of the three sites based on the best forecasting models.

Parameter	Mean	Median	Std	${℘}_{2.5}$	${℘}_{97.5}$	CD ${\;}^{a}$	Ineff. ${\;}^{b}$
Zuoying							
$\pi_{1}$	0.0019	0.0014	0.0018	0.0000	0.0066	0.4910	3.2333
$\pi_{2}$	0.0871	0.0879	0.0208	0.0439	0.1265	0.1386	5.1295
$\alpha_{0}$	0.0325	0.0305	0.0156	0.0073	0.0672	0.0982	2.9496
$\alpha_{1}$	0.6316	0.6312	0.0690	0.4951	0.7679	0.2377	3.4388
$\beta_{1}$	0.3787	0.3767	0.0645	0.2542	0.5052	0.4152	3.2421
$\gamma_{1}$	0.0459	0.0421	0.0285	0.0032	0.1095	0.8882	3.1974
Pingtung							
$\pi_{1}$	0.0018	0.0013	0.0017	0.0001	0.0065	0.7178	3.3460
$\pi_{2}$	0.1114	0.1115	0.0216	0.0705	0.1553	0.5319	2.9995
$\alpha_{0}$	0.0768	0.0749	0.0273	0.0303	0.1368	0.5904	3.3300
$\alpha_{1}$	0.5821	0.5825	0.0855	0.4211	0.7495	0.3520	3.5807
$\beta_{1}$	0.4145	0.4130	0.0881	0.2471	0.5837	0.5017	3.5029
$\gamma_{1}$	0.0073	0.0054	0.0066	0.0002	0.0243	0.0759	3.5047
Miaoli							
$\pi_{1}$	0.0059	0.0041	0.0060	0.0002	0.0223	0.4763	6.5837
$\pi_{2}$	0.3946	0.3942	0.0215	0.3529	0.4357	0.0630	3.5333
$\alpha_{0}$	0.0283	0.0252	0.0202	0.0014	0.0779	0.5110	3.7953
$\alpha_{1}$	0.6972	0.6955	0.0625	0.5805	0.8228	0.8103	3.4394
$\beta_{1}$	0.1312	0.1284	0.0667	0.0117	0.2633	0.1030	3.4457
$\gamma_{1}$	0.0200	0.0155	0.0166	0.0008	0.0613	0.1187	3.7250
$\gamma_{2}$	0.0479	0.0420	0.0338	0.0033	0.1282	0.2911	3.4330

^a^ CD: *p*-values of convergence diagnostic test. ^b^ Ineff.: inefficiency factors.

**Table 7:** Number of days and proportions of forecasted AQI levels and true AQI levels for Pingtung, Miaoli, and Zuoying.

	Pingtung					Miaoli					Zuoying			
**Fitted Model**	**0**	**1**	**2**	**3**		**0**	**1**	**2**	**3**		**0**	**1**	**2**	**3**
**True level ( $Y_{t}$ )**														
**# of days**	**98**	**164**	**82**	**21**		**152**	**191**	**21**	**1**		**125**	**134**	**84**	**22**
**Percentage (%)**	**26.8**	**44.9**	**22.5**	**5.8**		**41.6**	**52.3**	**5.8**	**0.3**		**34.2**	**36.7**	**23**	**6**
ZOBPAR ( $\hat{Y_{t}}$ )														
# of days	124	131	68	42		124	205	31	5		149	116	51	49
Percentage (%)	34.0	35.9	18.6	11.5		34.0	56.2	8.5	1.4		40.8	31.8	14.0	13.4
ZOBPARX 1 ( $\hat{Y_{t}}$ )														
# of days	140	117	60	48		133	195	21	16		130	132	53	50
Percentage (%)	38.4	32.1	16.4	13.2		36.4	53.4	5.8	4.4		35.6	36.2	14.5	13.7
ZOBPARX 2 ( $\hat{Y_{t}}$ )														
# of days	127	139	58	41		140	195	19	11		138	119	56	52
Percentage (%)	34.8	38.1	15.9	11.2		38.4	53.4	5.2	3.0		37.8	32.6	15.3	14.2
ZOBPARX 3 ( $\hat{Y_{t}}$ )														
# of days	132	120	56	57		114	219	20	12		129	132	59	45
Percentage (%)	36.2	32.9	15.3	15.6		31.2	60.0	5.5	3.3		35.3	36.2	16.2	12.3
ZOBPARX 4 ( $\hat{Y_{t}}$ )														
# of days	123	133	61	48		130	197	29	9		138	127	59	41
Percentage (%)	33.7	36.4	16.7	13.2		35.6	54.0	7.9	2.5		37.8	34.8	16.2	11.2

## Data Availability

The data presented in this study are available upon request from the corresponding author.
